# Chromatin Dynamics during Nucleotide Excision Repair: Histones on the Move

**DOI:** 10.3390/ijms130911895

**Published:** 2012-09-19

**Authors:** Salomé Adam, Sophie E. Polo

**Affiliations:** 1 Laboratory of Chromatin Dynamics, Curie Institute Research Centre, 75248 Paris Cedex 5, France; E-Mail: salome.adam@curie.fr; 2 Centre National de la Recherche Scientifique, Unité Mixte de Recherche 218, 75248 Paris Cedex 5, France

**Keywords:** chromatin, histone chaperone, histone dynamics, nucleosome remodeling factor, Nucleotide Excision Repair, ubiquitylation, UV

## Abstract

It has been a long-standing question how DNA damage repair proceeds in a nuclear environment where DNA is packaged into chromatin. Several decades of analysis combining *in vitro* and *in vivo* studies in various model organisms ranging from yeast to human have markedly increased our understanding of the mechanisms underlying chromatin disorganization upon damage detection and re-assembly after repair. Here, we review the methods that have been developed over the years to delineate chromatin alterations in response to DNA damage by focusing on the well-characterized Nucleotide Excision Repair (NER) pathway. We also highlight how these methods have provided key mechanistic insight into histone dynamics coupled to repair in mammals, raising new issues about the maintenance of chromatin integrity. In particular, we discuss how NER factors and central players in chromatin dynamics such as histone modifiers, nucleosome remodeling factors, and histone chaperones function to mobilize histones during repair.

## 1. Introduction

In the cell nucleus, DNA is packaged into chromatin, a complex nucleoprotein structure whose basic unit is the nucleosome [1]. The nucleosome core particle is composed of approximately 146 base pairs of DNA wrapped around an octamer of histone proteins comprising a (H3–H4)_2_ tetramer flanked by two H2A–H2B dimers [2]. Linker histones such as H1 and non-histone proteins also associate with the nucleosomal fiber, contributing to the formation of higher-order chromatin structures and nuclear domains [3,4]. Beyond this basic organization, the chromatin fiber shows variations in its compaction level and in its elementary components due to the existence of histone variants and post-translational modifications (reviewed in [5–8]). Altogether, these parameters provide an additional layer of information, potentially inherited through multiple cell generations, which controls gene expression and ultimately dictates cell function [3].

One of the key issues in the chromatin field is to understand how the information conveyed by chromatin is preserved while allowing all DNA metabolic activities (*i.e.*, DNA replication, transcription, and repair) that necessarily disorganize—at least transiently—chromatin structure. Several factors, including nucleosome remodeling factors, histone modifying enzymes, and histone chaperones, have emerged as critical players in chromatin dynamics (reviewed in [7,9–12]). Chromatin plasticity is particularly important during DNA repair as DNA damage occurs in an unscheduled manner and involves rearrangements of chromatin structure that prime chromatin for repair and help restore its integrity. These rearrangements, described in the Access/Prime-Repair-Restore model [13–16], involve the disorganization and subsequent re-organization of chromatin, the mechanisms of which are still incompletely understood.

Here, we review the methods that have been used to study histone dynamics during DNA repair and we present key findings from studies in mammalian cells that have contributed to build this model (extensive work conducted in other eukaryotic systems such as yeast is reviewed by others in this issue). More specifically, we focus on Nucleotide Excision Repair (NER), an evolutionarily conserved pathway that removes helix-distorting lesions such as those induced by UVC (UltraViolet C) light. This pathway has been extensively studied and its molecular players are well characterized (reviewed in [17]). Thus, it represents an attractive model that can be manipulated to address the issue of chromatin dynamics after DNA damage. In this review, we first describe the dynamic changes in chromatin structure that have been observed during NER. Then, we present our current knowledge of the mechanisms underlying these dynamics, with an emphasis on the role of specific histone modifications, nucleosome remodelers, and histone chaperones.

## 2. Dynamic Changes in Chromatin Structure during Nucleotide Excision Repair

**Figure 1:**
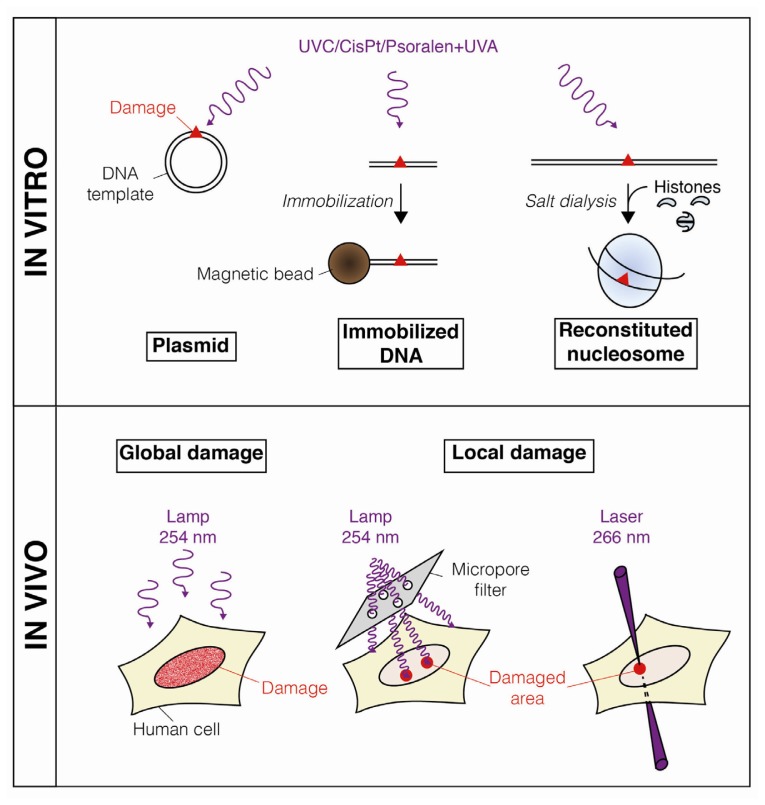
Methods for inducing DNA damage repaired by the Nucleotide Excision Repair (NER) pathway. When not chemically synthesized, DNA lesions (*i.e.*, pyrimidine dimers and bulky DNA adducts, red triangles) are generally induced by exposure to UV light and/or cross-linking agents (purple). Genotoxic treatment is applied on DNA templates (*in vitro*) or on cultured human cells (*in vivo*). Damaged DNA templates are further immobilized onto magnetic beads [19] or used to reconstitute nucleosome particles by salt dialysis [20,21]. Global cell irradiation with a UVC lamp generates DNA damage throughout the nucleus. Localized DNA damage is induced by irradiating cells with a UVC lamp through a micropore filter [22–24] or by focusing a UVC laser to specific sub-nuclear regions [25].

**Figure 2:**
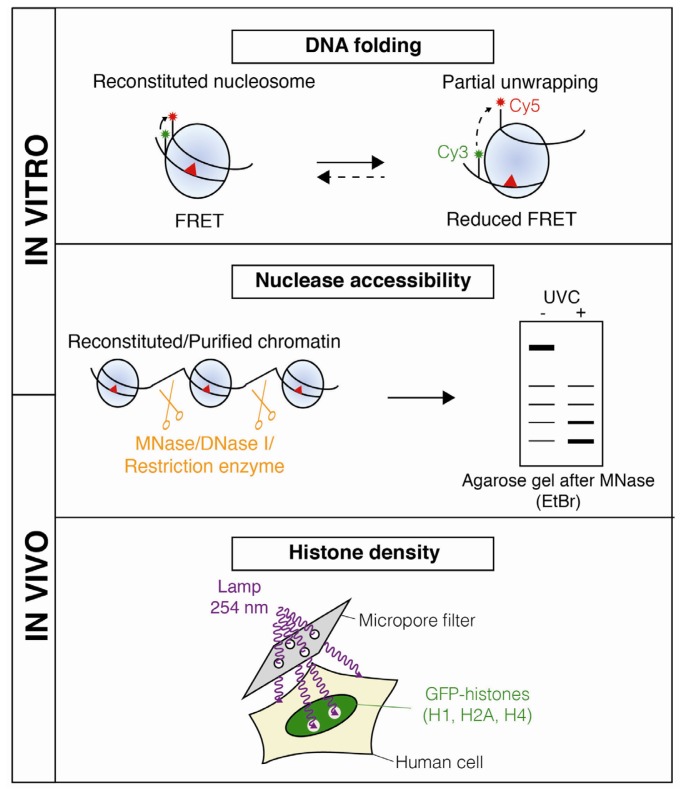
Methods for measuring chromatin disorganization upon UVC damage. Spontaneous changes in DNA folding in reconstituted nucleosomes containing UV photoproducts (red triangles) is measured by FRET (Fluorescence Resonance Energy Transfer) between the donor (Cy3) and acceptor (Cy5) fluorophores [21]. Chromatin rearrangements upon UVC damage can also be assessed by probing the accessibility of damaged DNA to nucleases (orange scissors), which is performed either on reconstituted chromatin (*in vitro*) or on chromatin purified from damaged cells (*in vivo*). The accumulation of small DNA fragments, indicative of disorganized chromatin (by nucleosome disruption and/or random positioning), is visualized by electrophoresis on an agarose gel stained with Ethidium Bromide (EtBr) [28,29,31]. *In vivo*, changes in histone density can be visualized by a decrease in fluorescent signal at sites of local UVC damage in cultured human cells expressing GFP-tagged histones (green) [32].

**Figure 3:**
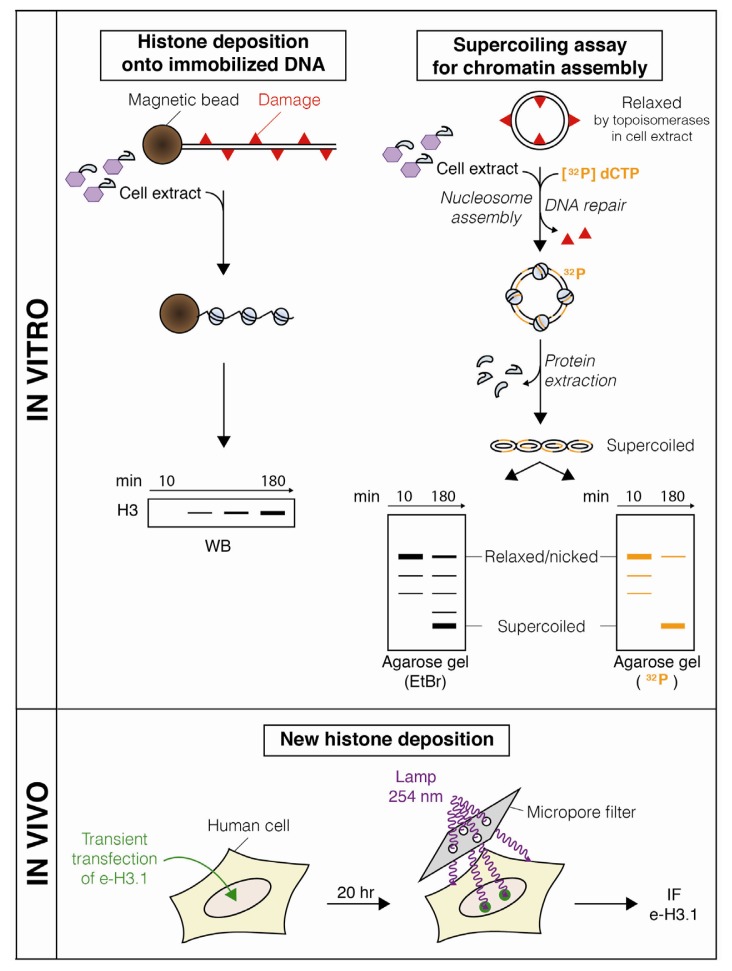
Methods for measuring chromatin restoration upon UVC damage. Deposition of histone proteins from a cell-free extract onto damaged DNA immobilized on magnetic beads is measured by western blotting (WB) against pulled-down histones [19,31]. Chromatin assembly coupled to NER can also be monitored *in vitro* by supercoiling assays using damaged plasmids mixed with extracts from human cells, xenopus eggs or drosophila embryos that are supplemented with a radioactive desoxyribonucleotide ([^32^P]dCTP which labels repair patches, orange). Within minutes, the plasmid is relaxed by topoisomerases present in the extracts. Nucleosome assembly introduces negative superhelical turns into the relaxed plasmid, which can be detected as faster migrating forms by electrophoresis on an agarose gel stained with Ethidium Bromide (EtBr, total DNA) or revealed by autoradiography (^32^P, repaired DNA) ([38,39], reviewed in [31]). *In vivo*, new histone deposition at sites of local UVC damage is visualized by immunofluorescence (IF) in cultured human cells transiently expressing epitope-tagged histones (e-H3.1, green) [40].

Over the past decades, a series of methods have been developed both *in vitro* and *in vivo* to assess histone and nucleosome dynamics coupled to NER (Figures 1–3), considerably increasing our understanding of chromatin rearrangements during the NER process.

### 2.1. DNA Damage-Induced Nucleosome Destabilization and Histone Mobilization

The NER pathway is involved in the repair of pyrimidine dimers and bulky DNA adducts that can be induced either in cultured cells or on DNA templates by irradiation with UVC light or treatment with cross-linking agents like Cisplatin (CisPt) or UVA-activated psoralen (Figure 1). Pyrimidine dimers can also be formed by chemical synthesis [18].

Early work studying how the NER pathway repairs UVC lesions on naked DNA revealed that, despite the short size of the repair patch (about 30 nucleotides in length) [26], access to at least 100 base pairs flanking the lesion was needed for the repair machinery to excise the damaged oligonucleotide [27]. These early observations suggested that, *in vivo*, the chromatin structure should be rearranged in order for repair factors to process UVC lesions. Formal proof of chromatin reorganization upon DNA damage was obtained by measuring chromatin accessibility to nucleases, which preferentially cleave the DNA between nucleosome particles (Figure 2). Pioneering experiments using partial MNase (Micrococcal Nuclease) digestion on chromatin purified from UVC-irradiated human fibroblasts [28] showed that chromatin regions undergoing NER present a transient increase in nuclease sensitivity. These initial observations were further confirmed by using other nucleases such as DNase I (DNA Nuclease 1) [29] or restriction enzymes [30].

Evidence of altered chromatin organization in the presence of UV photoproducts was recently obtained at the nucleosome scale. The FRET (Fluorescent Resonance Energy Transfer) technology has been adapted to follow spontaneous alterations in the folding of UV-damaged DNA in reconstituted nucleosomes (Figure 2). FRET efficiency is reduced in a UV dose-dependent manner showing that UV damaged nucleosomes remain partially unwrapped for longer times compared to undamaged nucleosomes. These data highlight that UV damage itself can cause changes in DNA wrapping around the histone octamer *in vitro*, which potentially facilitates access for repair machineries to DNA lesions *in vivo* [21].

In addition to the spontaneous dynamics of UV-damaged nucleosomes, active mechanisms also promote chromatin disorganization around UVC lesions, as recently visualized *in vivo* by locally irradiating cells through micropore filters (Figures 1 and 2). Human cells expressing fluorescently-labeled histones showed a decrease in fluorescence intensity at damage sites in an ATP (Adenosine TriPhosphate)-dependent manner, revealing that histone density is locally reduced by an active mechanism [32]. Whether this local loss of histones results from a complete disruption of damaged nucleosomes and/or nucleosome sliding away from the lesions is still to be determined.

**Figure 4:**
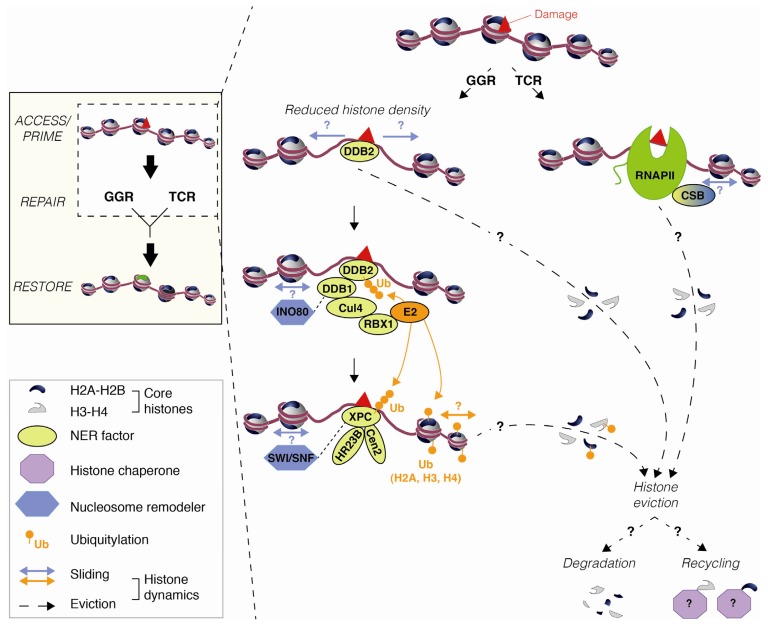
Histone dynamics at early steps of NER in mammals. The GGR (Global Genome Repair) factor DDB2 is recruited to the lesion (red triangle), where it promotes chromatin decompaction and histone displacement [32]. In conjunction with DDB1, Cul4 and RBX1, DDB2 forms an E3-ubiquitin ligase complex, which triggers ubiquitylation (orange) of several substrates. In particular, various core histones (H2A, H3 and H4) are ubiquitylated [48–53], which is thought to destabilize nucleosome structure and to promote histone loss from damaged chromatin [49,54]. The fate of histones displaced from damaged chromatin regions (degradation or recycling by histone chaperones) is still under investigation. In addition to a possible eviction of histones from damaged nucleosomes, histone dynamics during NER likely involves histone mobilization/sliding by nucleosome remodeling factors such as INO80, likely recruited via its interaction with DDB1 [55] and SWI/SNF, recruited concomitantly with the GGR factor XPC [56–59]. In addition to their association with repair factors, binding to DNA damage-induced histone modifications could be another mechanism for recruiting chromatin remodelers. During TCR (Transcription Coupled Repair), stalled RNAPII (green) is recognized by CSB, a SWI/SNF-like ATPase whose function in chromatin remodeling *in vivo* is still unclear [60].

Besides its likely role in giving access to repair machineries, histone eviction may also be a way to eliminate damaged histones (Figure 4). Indeed, UV irradiation can lead to the production of reactive oxygen species and free radicals via photosensitization mechanisms [33]. Histone proteins oxidized by such reactive molecules may then be targeted for degradation [34]. An alternative possibility is that histones removed from damaged chromatin regions are recycled by histone chaperones (Figure 4) and contribute to chromatin reorganization after DNA repair. Further studies on the fate of displaced histones will be required to understand how the original information conveyed by chromatin via histone variants and modifications can be preserved.

The extent of chromatin disorganization coupled to NER *in vivo* is also intriguing. Several lines of evidence suggest that it spreads far beyond the repair patch, at least up to 2 kilobases from the damage site [35], while another study reports that chromatin rearrangements extend over the whole nucleus following local UVC irradiation [36]. It is still unclear whether structural barriers to chromatin disorganization exist in the cell nucleus. As chromatin organizes itself in specific nuclear domains [3,4], it is conceivable that existing boundaries between these domains may limit spreading of chromatin rearrangements after DNA damage. The extent of chromatin disorganization after damage may also differ between euchromatin and more compact heterochromatin regions. To address these issues, a newly developed UVC laser micro-irradiation technique (Figure 1) would be interesting to use in the future as it provides a means to target the damage to specific sub-nuclear regions [25].

In conclusion, a large body of evidence implicates chromatin disorganization in NER, including damaged nucleosome destabilization and histone displacement, which raises the issue of how chromatin integrity can be preserved.

### 2.2. Nucleosome Restoration and New Histone Deposition Coupled to NER

The first evidence of chromatin restoration following NER came from nuclease digestion experiments on chromatin purified from UVC-damaged cells as described in the previous section [28,29]. These experiments indeed demonstrated that regions undergoing repair became progressively more nuclease resistant to finally present the same digestion profile as non-damaged chromatin, indicating restoration of nucleosomal arrays where nucleosomes occupy non-random positions. Furthermore, nucleosome restoration is complete with deposition of linker histone H1 [37] and re-establishment of a canonical DNase I footprint [29].

The coupling between NER and chromatin assembly was then further studied *in vitro* by using supercoiling assays (Figure 3). In these assays, UV or Cisplatin-damaged plasmids are mixed with cell-free extracts supplemented with radioactive desoxyribonucleotides to label repair patches, and analyzed by agarose gel electrophoresis and autoradiography. The accumulation of repaired supercoiled DNA molecules is indicative of nucleosome assembly coupled to NER [38,39].

**Figure 5:**
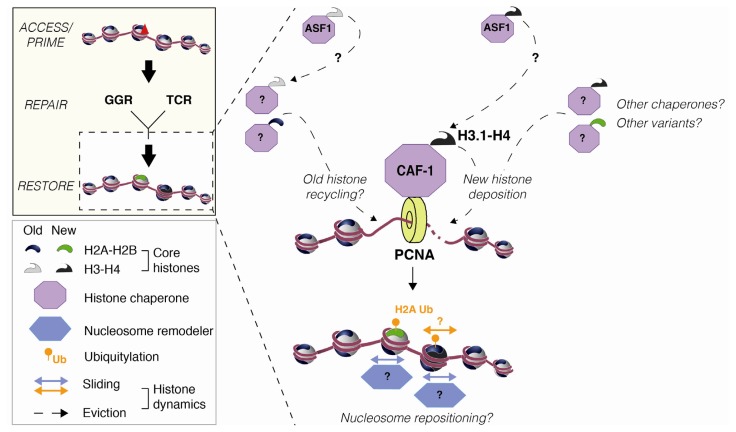
Histone dynamics at late steps of NER in mammals. Newly synthesized H3.1 histone variants in dimers with H4 (black) are deposited at UVC damage sites [40,41]. *De novo* histone deposition is coupled to repair synthesis via a direct interaction between the specific H3.1 histone chaperone CAF-1 (purple) and the polymerase sliding clamp PCNA (yellow) [67]. The chaperone ASF-1 may be a donor of new H3.1 for CAF-1 and/or contribute to old histone recycling. The involvement of other histone variants and other chaperones in the new histone deposition process is still to be determined. The contribution of old histone recycling is also an open issue. H2A is ubiquitylated (orange) in a CAF-1-dependent manner [52]. This modification, reported to destabilize nucleosome structure [54], might contribute in concert with nucleosome remodelers (blue) to re-position newly formed nucleosomes after repair.

The analysis of nucleosome dynamics during NER was then taken one step further by examining the role of specific histone variants. Indeed, most histones exist as distinct variants which differ in their amino-acid sequences, their expression profiles, and their timing and/or sites of incorporation into chromatin [5]. So far, the efforts towards investigating histone variant dynamics coupled to NER have been focused on the replicative H3.1 variant that is synthesized mostly in S phase and incorporated into nucleosomes in a DNA synthesis-coupled manner. *In vitro* experiments demonstrated that epitope-tagged H3.1 histone is deposited onto immobilized UV-damaged templates [41] (Figure 3). These data were then further strengthened *in vivo* in human cells transiently expressing Flag-HA-tagged H3.1. Upon local UVC irradiation, newly synthetized H3.1 histones accumulate at damage sites in a manner coupled to repair synthesis [40]. This study puts forward new H3.1 histone incorporation as critical in chromatin restoration after repair of UVC lesions (Figure 5).

Although it presumably helps restoring nucleosomal structure at damage sites, the incorporation of new histones challenges the maintenance of chromatin integrity. Indeed, newly synthesized soluble histones are known to bear post-translational modifications that differ from nucleosomal histones [42] and thus, deposition of new histones could lead to substantial changes in the chromatin landscape in repaired regions. Whether such changes are only transient or longer-term, leaving an imprint on chromatin, is an issue that clearly deserves further investigation. Notably, the dynamics of pre-existing histones and other variants also needs to be considered, and histone deposition at earlier steps in the NER process cannot be excluded. Further investigation of histone variant dynamics coupled to NER *in vivo* should now be possible by exploiting SNAP-tag-based pulse-chase imaging, a powerful technique that allows tracking new or old histones in live cells and quantifying their turnover [43–46].

Altogether, studies of chromatin dynamics coupled to NER reveal that chromatin undergoes dramatic changes in its organization during the repair process, involving nucleosome rearrangements and mobilization of histone proteins. Identifying the molecular players in these processes has been the focus of intense research, providing interesting mechanistic insights into histone dynamics coupled to NER, which we describe in the following section.

## 3. Mechanisms Underlying Histone Dynamics during Nucleotide Excision Repair

### 3.1. Chromatin Accessibility and Histone Post-Translational Modifications

Histone modifications play central roles in regulating chromatin dynamics not only during transcription but also in the context of DNA repair [7]. Historically, acetylation was the first histone post-translational modification shown to promote UV-damaged chromatin accessibility and to stimulate NER, as reported in yeast and mammalian cells (reviewed in [47] and by R. Waters and colleagues in this issue).

Studying how the NER machinery processes DNA lesions also revealed the importance of protein ubiquitylation in coordinating the NER response (for review, see [61]). Interestingly, in addition to various NER factors, H2A, H3 and H4 histones are ubiquitylated in the course of NER in mammals [48–53]. By examining H3 and H2A extractability from damaged chromatin *in vitro* and *in vivo* [49,54], histone ubiquitylation was shown to destabilize nucleosomal organization, suggesting that this modification could facilitate access to damaged chromatin *in vivo* by promoting histone displacement from damaged nucleosomes (Figure 4). Whether ubiquitylation alone is sufficient for increasing chromatin accessibility or if it acts as a signal for recruiting chromatin remodelers and/or histone chaperones is still to be determined.

The mechanisms for how this modification is established in response to UVC damage and coupled with NER are still under investigation. Several E3 ubiquitin ligases acting at different steps of the NER pathway have been identified as histone modifiers. First, by taking advantage of NER-deficient cell lines established from XPE (Xeroderma Pigmentosum E) patients, the E3 ubiquitin ligase complex RBX1 (Ring-BoX 1)-Cul4 (Cullin 4)-DDB1-DDB2 (DNA Damage Binding protein), a key player in UVC damage detection, was shown to ubiquitylate H2A *in vitro* and *in vivo* [48,50] (Figure 4). This complex is also involved in H3 and H4 ubiquitylation stimulated by UVC irradiation [49]. In addition, H2A was found to be ubiquitylated by the ubiquitin ligase RNF2 (Ring finger protein 2) in a manner dependent on the NER factor XPA [51]. The ubiquitin ligase RNF8 (Ring finger protein 8) also modifies H2A upon formation of singled stranded DNA, an NER intermediate resulting from lesion processing [53]. While the multiplicity of E3 ubiquitin ligases involved in modifying H2A complicates the analysis of its function in the NER pathway, it clearly underlines a critical role of this modified histone in this process. Finally, H2A ubiquitylation has been proposed to occur after repair synthesis and to be dependent on the H3.1 histone chaperone CAF-1 (Chromatin Assembly Factor 1) [52]. In this context, histone ubiquitylation, reported to destabilize nucleosomes, might help remodelers to reposition newly formed nucleosomes and could thus be an important player in chromatin restoration upon UVC irradiation (Figure 5). Future experiments will help clarify this issue and define the role of this modification in chromatin rearrangements coupled to early and late NER steps.

In conclusion, histone modifications by acetylation and ubiquitylation have emerged as key regulators of chromatin accessibility during NER. How they potentially crosstalk with other factors involved in chromatin dynamics such as remodelers and histone chaperones will be important to consider.

### 3.2. Nucleosome Mobilization by Chromatin Remodeling Factors

Chromatin remodelers use the energy of ATP hydrolysis to disrupt histone-DNA contacts, thus promoting nucleosome sliding, removal or exchange [9,10]. Remodeling factors were first identified as key regulators of gene expression, and it is only recently that their role in DNA damage response pathways has been investigated (reviewed in [62]).

In mammalian cells, both SWI/SNF (SWItch/Sucrose Non Fermentable) and INO80 (INOsitol requiring 80) remodeling factors stimulate NER as their down-regulation confers hypersensitivity to UVC, associated with inefficient removal of UV damage and impaired recruitment of early/intermediate NER factors [55–59]. The coupling between remodelers and NER factors is further supported by co-immunoprecipitation experiments revealing interactions between the SWI/SNF complex subunits BRG1 (Brahma-Related Gene 1) and SNF5/INI1 (Integrase Interactor 1) with XPC [57,59] and between INO80 and DDB1 [55] (Figure 4). Additionally, the first hints towards a possible involvement of other mammalian chromatin remodelers in NER started to emerge with members of the CHD (Chromodomain Helicase DNA binding protein) and ISWI (Imitation SWItch) families whose loss of function sensitizes cells to UVC irradiation [62,63].

However, the precise role of these remodelers at UV damage sites is still an open issue. Such factors likely help reorganize the chromatin structure for repair machineries to have access to DNA damage. While BRG1 and to a lesser extent INO80 were shown to increase chromatin accessibility upon global UV irradiation in human cells as revealed by MNase digestion profiles [55,57], it is not formally demonstrated that such factors actually promote nucleosome remodeling locally at DNA damage sites. Whether different remodelers fulfill distinct/complementary functions at damage sites (*i.e.*, nucleosome sliding *vs*. disruption) is another issue that warrants further investigation.

Besides well-defined chromatin remodelers, some NER factors also display nucleosome remodeling activity. In mammals, CSB (Cockayne Syndrome B), a NER factor involved in repair of UV damage on transcribed DNA strands, contains a SWI/SNF ATPase domain and was shown to remodel chromatin *in vitro*, but the relevance of this activity *in vivo* still needs to be addressed [60] (Figure 4). Additionally, a recent study revealed that the UV damage recognition factor DDB2 promotes chromatin decompaction and ATP-dependent histone displacement from sites of local UVC irradiation ([32], Figure 4). Notably, this function is independent of the E3 ubiquitin ligase activity of the CUL4-DDB complex and does not rely on SWI/SNF remodelers. The mechanisms underlying DDB2-mediated chromatin remodeling at damage sites are still unclear and most likely mediated by remodeling factor(s) yet to be identified.

Altogether, current studies mainly support a role for remodelers in promoting chromatin accessibility by moving histones/nucleosomes away from the damage site. Nevertheless, this does not exclude a possible function during chromatin restoration in cooperation with histone chaperones.

### 3.3. Histone Mobilization by Histone Chaperones

Histone chaperones are key players in histone metabolism involved in escorting histones and mobilizing them in and out of chromatin [11,12]. The list of known histone chaperones has been growing significantly, but only a few have been associated with the DNA damage response (reviewed in [16,64]) and CAF-1 is the only one with a well-described role in the context of NER in mammalian cells. CAF-1 is an evolutionarily conserved complex, initially identified by its ability to promote histone deposition coupled to DNA replication [65,66] and characterized later as a chaperone dedicated to the replicative H3.1 variant [41,45]. Notably, CAF-1 function is not restricted to replication, as it is recruited to DNA damage sites during late steps of NER as shown *in vitro* and in human cells [67–69]. Interestingly, consistent with a late recruitment in NER, CAF-1 is not required *per se* for efficient repair of UV lesions or for the recruitment of NER factors to damage in human cells [40]. Its function during NER was elucidated by a series of *in vitro* and *in vivo* experiments demonstrating that this histone chaperone promotes chromatin restoration coupled to NER by depositing newly synthesized H3.1 histones at damage sites in a repair-synthesis dependent manner [38,40,41] (Figure 5). The direct interaction of CAF-1 with the polymerase sliding clamp PCNA (Proliferating Cell Nuclear Antigen) provides molecular support for a coupling between chromatin re-assembly and DNA synthesis both during replication and repair [67,70].

In conclusion, the histone chaperone CAF-1 stands out as a key factor in chromatin restoration coupled to late NER via its ability to deposit new H3.1 histones at damage sites (Figure 5). The contribution of other histone chaperones to this process is still to be determined. In this respect, ASF-1 (Anti-Silencing Function 1) is an interesting candidate as it functions synergistically with CAF-1 to assemble nucleosomes during NER *in vitro* [71] and helps turning off the DNA damage checkpoint after UV irradiation both in yeast and mammalian cells [72,73]. It is thus possible that ASF-1 acts as a donor of new histones for CAF-1 in chromatin restoration coupled to NER (Figure 5). Another attractive possibility is that ASF1 could be involved in old histone recycling at damage sites (Figure 5) as described at the replication fork [74]. Future studies may also give more insights into the composition of nucleosomes formed upon repair-coupled chromatin restoration by determining whether histone variants other than H3.1 get deposited at NER sites.

## 4. Conclusions and Open Issues

As reviewed here and summarized in the Access/Prime-Repair-Restore model [13–16], the NER response induces dramatic chromatin structural changes, which involve histone modification and mobilization at various steps of the NER pathway. The underlying molecular mechanisms and the functional relevance of chromatin dynamics during NER are topics of intense research. Chromatin rearrangements at early stages of NER expose DNA damage to NER machineries and may also facilitate the removal of damaged histone proteins. Such disorganization of chromatin most likely results from both histone eviction by nucleosome disruption and nucleosome sliding away from DNA lesions. It is achieved by the concerted action of NER factors involved in UV damage detection, histone modifiers and chromatin remodeling factors (Figure 4). After DNA damage processing, chromatin re-assembly involves a coupling between late NER factors and histone chaperones incorporating new H3.1 histones at damage sites (Figure 5).

Nevertheless, many aspects of chromatin dynamics during NER are still unresolved. In particular, the roles of histone modifiers, nucleosome remodelers, and histone chaperones during NER have been mostly considered separately and at specific repair steps. Thus, a future challenge in the field will be to understand how the different players involved in damaged chromatin dynamics work together in a coordinated fashion. Distinct players might also be involved depending on the type of damage inflicted. It would thus be interesting to analyze potential differences in histone dynamics among the several DNA repair pathways. Another critical issue will be to determine whether and how chromatin disorganization and restoration are coupled. This may help to understand how the information conveyed by chromatin organization is preserved upon repair of DNA damage. As discussed above, new histone incorporation at damage sites is likely to modify, at least transiently, the information encoded by chromatin structure via alterations in patterns of histone variants and modifications. Since these chromatin marks play critical roles in transcription regulation, any change at this level may impact on the expression of the genes in the damaged chromatin region. To assess the extent of such changes, it will be important to determine the relative proportions of new and old histones involved in chromatin reorganization following NER. How long these new histones persist at repair sites and whether they could mark chromatin regions that have been damaged is another important question that needs to be addressed.

Finally, the impact of chromatin higher-order structures on NER and the dynamics and functions of non histone proteins during this process would be interesting to explore in future studies. Indeed, the importance of non histone chromatin proteins was recently revealed in the response to DNA double-strand breaks with several reports underlining how repair of DNA damage proceeds in compact heterochromatin domains (reviewed in [16]). The role of non histone chromatin-associated proteins in NER started to be analyzed in human cells and *C. elegans*, with evidence for an involvement of HP1 family members (Heterochromatin Protein 1) in the response to UV damage [75]. How silenced heterochromatin-like structures impede the NER process has been addressed only in yeast so far [76,77]. Future work should shed light on the interplay between NER and chromatin, to decipher not only how NER modulates chromatin structure but also how distinct chromatin organization levels differentially regulate NER.

## References

[1] Kornberg R.D. (1977). Structure of chromatin. Annu. Rev. Biochem.

[2] Luger K., Mäder A.W., Richmond R.K., Sargent D.F., Richmond T.J. (1997). Crystal structure of the nucleosome core particle at 2.8 A resolution. Nature.

[3] Probst A.V., Dunleavy E., Almouzni G. (2009). Epigenetic inheritance during the cell cycle. Nat. Rev. Mol. Cell Biol.

[4] Li G., Reinberg D. (2011). Chromatin higher-order structures and gene regulation. Curr. Opin. Genet. Dev.

[5] Talbert P.B., Henikoff S. (2010). Histone variants—Ancient wrap artists of the epigenome. Nat. Rev. Mol. Cell Biol.

[6] Talbert P.B., Ahmad K., Almouzni G., Ausió J., Berger F., Bhalla P.L., Bonner W.M., Cande W.Z., Chadwick B.P., Chan S.W.L. (2012). A unified phylogeny-based nomenclature for histone variants. Epigenetics Chromatin.

[7] Bannister A.J., Kouzarides T. (2011). Regulation of chromatin by histone modifications. Cell Res.

[8] Luger K., Dechassa M.L., Tremethick D.J. (2012). New insights into nucleosome and chromatin structure: An ordered state or a disordered affair?. Nat. Rev. Mol. Cell Biol.

[9] Flaus A., Owen-Hughes T. (2011). Mechanisms for ATP-dependent chromatin remodelling: The means to the end. FEBS J.

[10] Hargreaves D.C., Crabtree G.R. (2011). ATP-dependent chromatin remodeling: Genetics, genomics and mechanisms. Cell Res.

[11] De Koning L., Corpet A., Haber J.E., Almouzni G. (2007). Histone chaperones: An escort network regulating histone traffic. Nat. Struct. Mol. Biol.

[12] Avvakumov N., Nourani A., Côté J. (2011). Histone chaperones: Modulators of chromatin marks. Mol. Cell.

[13] Smerdon M.J. (1991). DNA repair and the role of chromatin structure. Curr. Opin. Cell Biol.

[14] Green C.M., Almouzni G. (2002). When repair meets chromatin. First in series on chromatin dynamics. EMBO Rep.

[15] Groth A., Rocha W., Verreault A., Almouzni G. (2007). Chromatin challenges during DNA replication and repair. Cell.

[16] Soria G., Polo S.E., Almouzni G. (2012). Prime, repair, restore: The active role of chromatin in the DNA damage response. Mol. Cell.

[17] Nouspikel T. (2009). DNA repair in mammalian cells: Nucleotide excision repair: Variations on versatility. Cell. Mol. Life Sci.

[18] Taylor J.S., Brockie I.R., O’Day C.L. (1987). A building block for the sequence-specific introduction of *cis*-syn thymine dimers into oligonucleotides. Solid-phase synthesis of TpT[c,s]pTpT. J. Am. Chem. Soc.

[19] Mello J.A., Moggs J.G., Almouzni G. (2006). Analysis of DNA repair and chromatin assembly *in vitro* using immobilized damaged DNA substrates. Methods Mol. Biol.

[20] Ura K., Araki M., Saeki H., Masutani C., Ito T., Iwai S., Mizukoshi T., Kaneda Y., Hanaoka F. (2001). ATP-dependent chromatin remodeling facilitates nucleotide excision repair of UV-induced DNA lesions in synthetic dinucleosomes. EMBO J.

[21] Duan M.-R., Smerdon M.J. (2010). UV damage in DNA promotes nucleosome unwrapping. J. Biol. Chem.

[22] Katsumi S., Kobayashi N., Imoto K., Nakagawa A., Yamashina Y., Muramatsu T., Shirai T., Miyagawa S., Sugiura S., Hanaoka F. (2001). *In situ* visualization of ultraviolet-light-induced DNA damage repair in locally irradiated human fibroblasts. J. Invest. Dermatol.

[23] Moné M.J., Volker M., Nikaido O., Mullenders L.H., van Zeeland A.A., Verschure P.J., Manders E.M., van Driel R. (2001). Local UV-induced DNA damage in cell nuclei results in local transcription inhibition. EMBO Rep.

[24] Guerrero-Santoro J., Levine A.S., Rapić-Otrin V. (2011). Co-localization of DNA repair proteins with UV-induced DNA damage in locally irradiated cells. Methods Mol. Biol.

[25] Dinant C., de Jager M., Essers J., van Cappellen W.A., Kanaar R., Houtsmuller A.B., Vermeulen W. (2007). Activation of multiple DNA repair pathways by sub-nuclear damage induction methods. J. Cell Sci.

[26] Edenberg H., Hanawalt P. (1972). Size of repair patches in the DNA of ultraviolet-irradiated HeLa cells. Biochim. Biophys. Acta.

[27] Huang J.C., Sancar A. (1994). Determination of minimum substrate size for human excinuclease. J. Biol. Chem.

[28] Smerdon M.J., Lieberman M.W. (1978). Nucleosome rearrangement in human chromatin during UV-induced DNA-repair synthesis. Proc. Natl. Acad. Sci. USA.

[29] Smerdon M.J., Lieberman M.W. (1980). Distribution within chromatin of deoxyribonucleic acid repair synthesis occurring at different times after ultraviolet radiation. Biochemistry.

[30] Gong F., Fahy D., Smerdon M.J. (2006). Rad4-Rad23 interaction with SWI/SNF links ATP-dependent chromatin remodeling with nucleotide excision repair. Nat. Struct. Mol. Biol.

[31] Gérard A., Polo S.E., Roche D., Almouzni G. (2006). Methods for studying chromatin assembly coupled to DNA repair. Meth. Enzymol.

[32] Luijsterburg M.S., Lindh M., Acs K., Vrouwe M.G., Pines A., van Attikum H., Mullenders L.H., Dantuma N.P. (2012). DDB2 promotes chromatin decondensation at UV-induced DNA damage. J. Cell Biol.

[33] Pattison D.I., Davies M.J. (2006). Actions of ultraviolet light on cellular structures. Experientia Supplementum.

[34] Bader N., Grune T. (2006). Protein oxidation and proteolysis. Biol. Chem.

[35] Mathis G.A., Althaus F.R. (1990). Isolation of 8-methoxypsoralen accessible DNA domains from chromatin of intact cells. Cell Biol. Toxicol.

[36] Rubbi C.P., Milner J. (2003). p53 is a chromatin accessibility factor for nucleotide excision repair of DNA damage. EMBO J.

[37] Smerdon M.J., Watkins J.F., Lieberman M.W. (1982). Effect of histone H1 removal on the distribution of ultraviolet-induced deoxyribonucleic acid repair synthesis within chromatin. Biochemistry.

[38] Gaillard P.H., Martini E.M., Kaufman P.D., Stillman B., Moustacchi E., Almouzni G. (1996). Chromatin assembly coupled to DNA repair: A new role for chromatin assembly factor I. Cell.

[39] Gaillard P.H., Moggs J.G., Roche D.M., Quivy J.P., Becker P.B., Wood R.D., Almouzni G. (1997). Initiation and bidirectional propagation of chromatin assembly from a target site for nucleotide excision repair. EMBO J.

[40] Polo S.E., Roche D., Almouzni G. (2006). New histone incorporation marks sites of UV repair in human cells. Cell.

[41] Tagami H., Ray-Gallet D., Almouzni G., Nakatani Y. (2004). Histone H3.1 and H3.3 complexes mediate nucleosome assembly pathways dependent or independent of DNA synthesis. Cell.

[42] Loyola A., Bonaldi T., Roche D., Imhof A., Almouzni G. (2006). PTMs on H3 variants before chromatin assembly potentiate their final epigenetic state. Mol. Cell.

[43] Jansen L.E.T., Black B.E., Foltz D.R., Cleveland D.W. (2007). Propagation of centromeric chromatin requires exit from mitosis. J. Cell Biol.

[44] Dunleavy E.M., Almouzni G., Karpen G.H. (2011). H3.3 is deposited at centromeres in S phase as a placeholder for newly assembled CENP-A in G_1_ phase. Nucleus.

[45] Ray-Gallet D., Woolfe A., Vassias I., Pellentz C., Lacoste N., Puri A., Schultz D.C., Pchelintsev N.A., Adams P.D., Jansen L.E.T. (2011). Dynamics of histone h3 deposition *in vivo* reveal a nucleosome gap-filling mechanism for h3.3 to maintain chromatin integrity. Mol. Cell.

[46] Bodor D.L., Rodríguez M.G., Moreno N (2012). Analysis of protein turnover by quantitative SNAP-based pulse-chase imaging. Curr. Protoc. Cell Biol.

[47] Reed S.H. (2011). Nucleotide excision repair in chromatin: Damage removal at the drop of a HAT. DNA Repair.

[48] Kapetanaki M.G., Guerrero-Santoro J., Bisi D.C., Hsieh C.L., Rapić-Otrin V., Levine A.S. (2006). The DDB1-CUL4ADDB2 ubiquitin ligase is deficient in xeroderma pigmentosum group E and targets histone H2A at UV-damaged DNA sites. Proc. Natl. Acad. Sci. USA.

[49] Wang H., Zhai L., Xu J., Joo H.-Y., Jackson S., Erdjument-Bromage H., Tempst P., Xiong Y., Zhang Y. (2006). Histone H3 and H4 ubiquitylation by the CUL4-DDB-ROC1 ubiquitin ligase facilitates cellular response to DNA damage. Mol. Cell.

[50] Guerrero-Santoro J., Kapetanaki M.G., Hsieh C.L., Gorbachinsky I., Levine A.S., Rapić-Otrin V. (2008). The cullin 4B-based UV-damaged DNA-binding protein ligase binds to UV-damaged chromatin and ubiquitinates histone H2A. Cancer Res.

[51] Bergink S., Salomons F.A., Hoogstraten D., Groothuis T.A.M., de Waard H., Wu J., Yuan L., Citterio E., Houtsmuller A.B., Neefjes J. (2006). DNA damage triggers nucleotide excision repair-dependent monoubiquitylation of histone H2A. Genes Dev.

[52] Zhu Q., Wani G., Arab H.H., El-Mahdy M.A., Ray A., Wani A.A. (2009). Chromatin restoration following nucleotide excision repair involves the incorporation of ubiquitinated H2A at damaged genomic sites. DNA Repair.

[53] Marteijn J.A., Bekker-Jensen S., Mailand N., Lans H., Schwertman P., Gourdin A.M., Dantuma N.P., Lukas J., Vermeulen W. (2009). Nucleotide excision repair-induced H2A ubiquitination is dependent on MDC1 and RNF8 and reveals a universal DNA damage response. J. Cell Biol.

[54] Lan L., Nakajima S., Kapetanaki M.G., Hsieh C.L., Fagerburg M., Thickman K., Rodriguez-Collazo P., Leuba S.H., Levine A.S., Rapić-Otrin V. (2012). Monoubiquitinated H2A destabilizes photolesion-containing nucleosomes with the concomitant release of the UV-damaged DNA-binding protein E3 ligase. J. Biol. Chem.

[55] Jiang Y., Wang X., Bao S., Guo R., Johnson D.G., Shen X., Li L. (2010). INO80 chromatin remodeling complex promotes the removal of UV lesions by the nucleotide excision repair pathway. Proc. Natl. Acad. Sci. USA.

[56] Gong F., Fahy D., Liu H., Wang W., Smerdon M.J. (2008). Role of the mammalian SWI/SNF chromatin remodeling complex in the cellular response to UV damage. Cell Cycle.

[57] Zhao Q., Wang Q.-E., Ray A., Wani G., Han C., Milum K., Wani A.A. (2009). Modulation of nucleotide excision repair by mammalian SWI/SNF chromatin-remodeling complex. J. Biol. Chem.

[58] Zhang L., Zhang Q., Jones K., Patel M., Gong F. (2009). The chromatin remodeling factor BRG1 stimulates nucleotide excision repair by facilitating recruitment of XPC to sites of DNA damage. Cell Cycle.

[59] Ray A., Mir S.N., Wani G., Zhao Q., Battu A., Zhu Q., Wang Q.-E., Wani A.A. (2009). Human SNF5/INI1, a component of the human SWI/SNF chromatin remodeling complex, promotes nucleotide excision repair by influencing ATM recruitment and downstream H2AX phosphorylation. Mol. Cell. Biol.

[60] Citterio E., van den Boom V., Schnitzler G., Kanaar R., Bonte E., Kingston R.E., Hoeijmakers J.H., Vermeulen W. (2000). ATP-dependent chromatin remodeling by the Cockayne syndrome B DNA repair-transcription-coupling factor. Mol. Cell. Biol.

[61] Nouspikel T. (2011). Multiple roles of ubiquitination in the control of nucleotide excision repair. Mech. Ageing Dev.

[62] Lans H., Marteijn J.A., Vermeulen W. (2012). ATP-dependent chromatin remodeling in the DNA-damage response. Epigenetics Chromatin.

[63] Rajagopalan S., Nepa J., Venkatachalam S. (2012). Chromodomain helicase DNA-binding protein 2 affects the repair of X-ray and UV-induced DNA damage. Environ. Mol. Mutagen.

[64] Ransom M., Dennehey B.K., Tyler J.K. (2010). Chaperoning histones during DNA replication and repair. Cell.

[65] Smith S., Stillman B. (1989). Purification and characterization of CAF-I, a human cell factor required for chromatin assembly during DNA replication *in vitro*. Cell.

[66] Stillman B. (1986). Chromatin assembly during SV40 DNA replication *in vitro*. Cell.

[67] Moggs J.G., Grandi P., Quivy J.P., Jónsson Z.O., Hübscher U., Becker P.B., Almouzni G. (2000). A CAF-1-PCNA-mediated chromatin assembly pathway triggered by sensing DNA damage. Mol. Cell. Biol.

[68] Martini E., Roche D.M., Marheineke K., Verreault A., Almouzni G. (1998). Recruitment of phosphorylated chromatin assembly factor 1 to chromatin after UV irradiation of human cells. J. Cell Biol.

[69] Green C.M., Almouzni G. (2003). Local action of the chromatin assembly factor CAF-1 at sites of nucleotide excision repair *in vivo*. EMBO J.

[70] Shibahara K., Stillman B. (1999). Replication-dependent marking of DNA by PCNA facilitates CAF-1-coupled inheritance of chromatin. Cell.

[71] Mello J.A., Silljé H.H.W., Roche D.M.J., Kirschner D.B., Nigg E.A., Almouzni G. (2002). Human Asf1 and CAF-1 interact and synergize in a repair-coupled nucleosome assembly pathway. EMBO Rep.

[72] Kim J.-A., Haber J.E. (2009). Chromatin assembly factors Asf1 and CAF-1 have overlapping roles in deactivating the DNA damage checkpoint when DNA repair is complete. Proc. Natl. Acad. Sci. USA.

[73] Battu A., Ray A., Wani A.A. (2011). ASF1A and ATM regulate H3K56-mediated cell-cycle checkpoint recovery in response to UV irradiation. Nucleic Acids Res.

[74] Groth A., Corpet A., Cook A.J.L., Roche D., Bartek J., Lukas J., Almouzni G. (2007). Regulation of replication fork progression through histone supply and demand. Science.

[75] Luijsterburg M.S., Dinant C., Lans H., Stap J., Wiernasz E., Lagerwerf S., Warmerdam D.O., Lindh M., Brink M.C., Dobrucki J.W. (2009). Heterochromatin protein 1 is recruited to various types of DNA damage. J. Cell Biol.

[76] Livingstone-Zatchej M., Marcionelli R., Möller K., de Pril R., Thoma F. (2003). Repair of UV lesions in silenced chromatin provides *in vivo* evidence for a compact chromatin structure. J. Biol. Chem.

[77] Irizar A., Yu Y., Reed S.H., Louis E.J., Waters R. (2010). Silenced yeast chromatin is maintained by Sir2 in preference to permitting histone acetylations for efficient NER. Nucleic Acids Res.

